# Getting to the start line: how bumblebees and honeybees are visually guided towards their first floral contact

**DOI:** 10.1007/s00040-014-0366-2

**Published:** 2014-09-05

**Authors:** L. L. Orbán, C. M. S. Plowright

**Affiliations:** 1 School of Psychology, University of Ottawa, Ottawa, Canada; 2 Present Address: Department of Psychology, Kwantlen Polytechnic University, 12666 72nd Avenue, Surrey, BC Canada

**Keywords:** Visual recognition, Bumblebees, Honeybees, Innate, Unlearned behaviour, Flower-naïve

## Abstract

Much of the literature on foraging behaviour in bees focuses on what they learn after they have had rewarded experience with flowers. This review focuses on how honeybees and bumblebees are drawn to candidate food sources in the first place: the foundation on which learning is built. Prior to rewarded foraging experience, flower-naïve bumblebees and honeybees rely heavily on visual cues to discover their first flower. This review lists methodological issues that surround the study of flower-naïve behaviour and describes technological advances. The role of distinct visual properties of flowers in attracting bees is considered: colour, floral size, patterning and social cues. The research reviewed is multi-disciplinary and takes the perspectives of both the bees and the plants they visit. Several avenues for future research are proposed.

## Introduction

How do bees first find flowers? To behavioural ecologists, the question itself may seem perplexing: finding flowers is just what bees do. That bees are well designed to exploit floral resources is so self-evident that at first glance it may seem as if there is nothing to explain. Indeed, much of the research on foraging behaviour concerns what bees do, and how they do it, after they have had their first rewarded experience on flowers (see reviews by Gould, 1990; Bitterman, 1996; Chittka and Thomson, 2001; Menzel, 2001; Raine et al., 2006; Benard et al., 2006; Giurfa, 2007; Dukas, 2008; Goulson, 2010; Avarguès-Weber et al., 2011; Dyer, 2012). There is comparatively less research on what they do before: when workers leave their colony for the first time, having never yet encountered a flower, how do they identify candidate food sources? This review is centred on how bees are directed to their first floral contact where pollen and nectar rewards begin to shape their motor responses into efficient food-directed behavioural sequences: how bees get to the start line of their foraging careers.

If workers in eusocial species fail to find food, especially at the beginning of colony cycle, not only are a few individuals placed at risk, but the whole colony could fail to thrive or die out altogether. This review focuses on honeybees (*Apis* spp. L., 1758) and bumblebees (*Bombus* spp. Latreille, 1802). They are central place foragers that contribute to the nutrition of the entire colony. In addition, the visual processing in these Hymenopterans has been exceptionally well documented (Dyer, 2012)—there is a substantial body of literature to use in eventual comparisons in future research between behaviours before and after the first floral reward.

The question of what draws bees to potential sources of nectar and pollen may be of interest not only to insect behaviourists but also to pollination ecologists: this paper approaches the problem from the perspectives of the problems faced by insects and those faced by the plants they visit. Plants “advertise” themselves (Dafni et al., 2005) and incur high costs in doing so (Primack and Hall, 1990) but are, nonetheless, frequently subject to pollination deficits. Growers of insect-pollinated field crops such as blueberries and cranberries routinely pay for commercial pollination services to improve crop quality and yield (Free, 1993; Velthuis and van Doorn, 2005). Given the worldwide declines in bumblebees (Goulson et al., 2008; Williams and Osborne, 2009), the competition amongst flower species to attract pollinators may be on the rise. As noted by Buchmann and Nabhan (1996, p. 258): “Fewer pollinators ultimately mean fewer plants”, and understanding their pollination ecology will be critical to protect them from extinction.

The terminology regarding our subject area is fraught with difficulties in interpretation. The term “innate” (or a synonym, “instinctive”), though it persists in the biological literature, is problematic (Bateson, 1984; Oyama, 2000; Scholz, 2002; Bateson and Mameli, 2007; Mameli and Bateson, 2006, 2011) because it can take on several non-interchangeable meanings (e.g. adaptive, unmodifiable, inborn, hardwired, unlearned, species-specific, etc.). What is worse, evidence for one meaning can too easily be mistaken as entailing evidence for the other (Bateson and Gluckman, 2011). One possible solution is to use the term “pre-functional” (Hogan, 1994)—in our case, the behaviour that occurs prior to functional experience with flowers. A similar tack is to characterize the bees themselves as “flower-naïve” (Giurfa et al., 1995) or “foraging-naïve” (Milet-Pinheiro et al., 2012). We will adopt these expressions because they have the advantage that they avoid any implication that no experience whatsoever is necessary for the development of behaviour.

This review focuses only on visual cues that are attractive to flower-naïve honeybees and bumblebees though in nature, odour cues are almost certainly important as well. In bumblebees, workers take advantage of floral odours that are brought into the colony by others (Dornhaus and Chittka, 1999). The role of odour cues in isolation of, and in combination with, visual cues has been documented for solitary bee species (*Chelostoma rapunculi* (Lepeletier, 1841)): the relative importance of these cues changes with experience (Milet-Pinheiro et al., 2012). The use of various cues also depends on availability: bumblebees (*Bombus impatiens* Cresson, 1863) can forage in complete darkness (Chittka et al., 1999). The olfactory preferences of honeybees are reviewed by Riffell (2011). The chemical ecology and evolution of bee–flower interactions are reviewed by Dötterl and Vereecken (2010). Multi-sensory integration in bees is reviewed by Leonard and Masek (2014).

The overriding question in this paper is not new. It can be traced to the writings of Manning (1956, p. 198) (…“it is necessary for a plant to attract bees in the first place, before they are ‘aware’ of the food supply…”) and Free and Butler (1959, p. 106) (“Little work has been done to discover those features of flowers to which bees react on their very first foraging flights, and such an investigation would be well worth undertaking”). Giurfa et al. (1995) trace the question back to none other than Charles Darwin (1876). What is new is that now there are some answers. We begin with methodological considerations, follow with an examination of the role of various visual cues that have been investigated and conclude with suggestions for future research. We draw on the literature from perception, neuroscience, ecology and computational science. The benefits of a multi-disciplinary approach that integrates functional questions from biology with mechanistic questions from psychology have been delineated by Dukas (1998, 2004), Chittka and Thomson (2001), Dukas and Ratcliffe (2009) and Shettleworth (2010).

## Methodological issues

Though bees can be tracked in the field over long distances using harmonic radar (Osborne et al., 1999), it remains, as noted by Lunau and Maier (1995), methodologically intractable to determine the first flower choice of bees that are known to be flower-naïve. Accordingly, most of the research is conducted in the lab where the history of individual workers is known and the floral options can be controlled. Below we describe some of the standard procedures that have been used to investigate floral preferences of flower-naïve bees and highlight some of the methodological pitfalls. This section is intended as a guide to navigating the literature and as a list of experimental design considerations for use in future research.

### Pre-training

Workers that have had foraging experience in the lab typically fly directly to the source of food and return reliably. In contrast, the flight paths of flower-naïve bumblebees are typically meandering and it can take hours and even days before they alight on any artificial patterns. Indeed, on their first flights, the task of learning landmarks and the characteristics of their nest entrance (Hempel de Ibarra et al., 2009) may possibly take precedence over foraging. Even in greenhouses where there is little else but rows of tomato flowers, bumblebees can take 2–4 days before foraging reliably on the flowers (Asada and Ono, 1996). In a flight cage in our lab, the times in between first leaving a colony and landing on one of two artificial flowers for a sample of almost 200 bumblebees were distributed with a mode of within 1 day, but a median of 11 days (Orbán, 2013, unpubl. data). To circumvent this problem, bees are sometimes trained to ostensibly neutral patterns such as black discs, white discs (Rodríguez et al., 2004) or checkerboards (Lehrer et al., 1995) and subsequently tested for their preferences of new unrewarded patterns. This practice may be innocuous when studying colour preferences: bees do not generalize their experience from pre-training with one colour to testing on others, as long as the colours seem very different to them (Gumbert, 2000). Nonetheless, explicit tests of the effects of pre-training on subsequent pattern choice have shown differences in the behaviour of untrained (flower-naïve) and pre-trained (not-so-naïve) bumblebees (Séguin and Plowright, 2008; Plowright et al., 2011). Hence, the untested assumption that pre-training is neutral or unrelated to the test of floral preferences is tenuous.

### Choices

Strictly speaking, a bee is flower-naïve for its first choice, but not for its second. It is an empirical matter, however, whether the first few unrewarded choices differ from the first. There is habituation of unlearned preferences: preferences wane between testing sessions in the face of repeated exposure to patterns that offer no reward (Simonds and Plowright, 2004), but resurface again after time (Plowright et al., 2006). Over periods of prolonged testing on unrewarding patterns, where bees are free to return to and from the flight cage and their colony, increases and decreases in preferences oscillate (Orbán and Plowright, 2013). Within a testing session consisting of a series of unrewarded choices made upon the first trip away from the colony, however, we have found little or no change within short sessions of 16–20 choices (Plowright et al., 2011, 2013).

One issue that remains unresolved is the effect of the number of floral options presented. Even with a single flower, there is a choice to accept or reject it. With two flowers, an apparent preference for one flower can be the result of an avoidance of the other: preferences are relative. Offering three options would further complicate the situation. In the animal behaviour literature, the preference of one stimulus over the other can be affected in non-trivial ways by the introduction of a third option (Bateson, 2004). That this may be a real concern in our area is suggested by the work of Shafir (1994) who demonstrated intransitive preferences in honeybees (*Apis mellifera* L., 1758): in a series of binary choices that varied in the depth and volume of sucrose-water delivered, honeybees preferred A to B, B to C, C to D, but D to A.

### Measures of preference

**Fig. 1:**
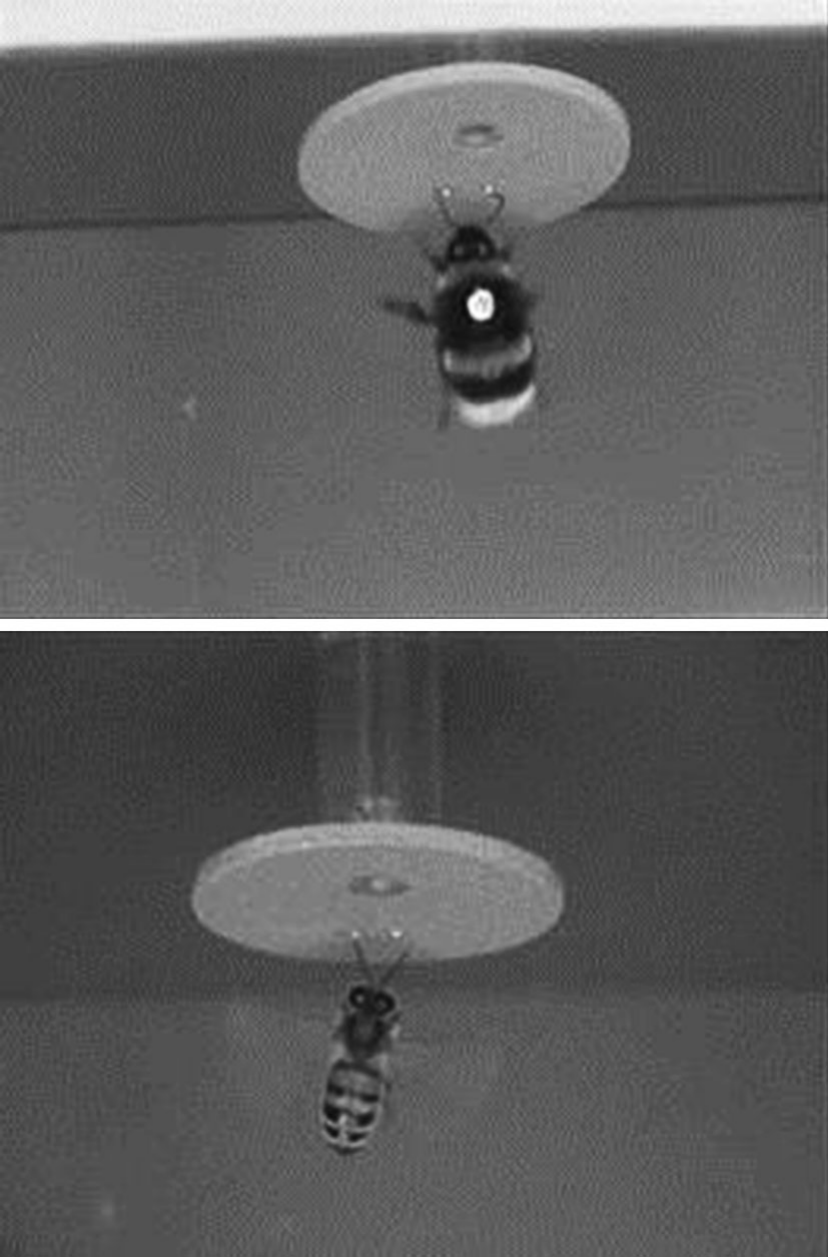
Antennal reactions towards floral guides (*two dots*) by *Bombus terrestris* (*above*) and *Apis mellifera* (*below*). Photograph from Lunau et al. (2009). Reprinted with kind permission from Springer Science and Business Media

Choice behaviour can be measured in multiple ways, differing in the level of apparent commitment to a floral stimulus by the bee: approach within a specified distance (e.g. 2 cm; Goulson et al., 2007); entering a corridor, in which a pattern is contained, in a maze (Simonds and Plowright, 2004; Séguin and Plowright, 2008); antennal contact with a test pattern (Pohl et al., 2008; Lunau et al., 2009)—see Fig. 1 for an illustration with honeybees and bumblebees; landing on a test pattern (Leonard and Papaj, 2011); floral exploration as defined by walking into an artificial flower (Orbán and Plowright, 2013); or probing (Daumer, 1958). Even finer gradations can be achieved—see Evangelista et al. (2010) for details on the moments before touchdown in honeybees. Some of these different behaviours are sometimes lumped together and discussed as ‘preference’ even though floral choice consists of a series of sequential decisions that are not necessarily governed by the same parameters (Lunau, 1992; Lunau et al., 2006).

### Individual differences

Individual differences in the behaviour of eusocial insects are pronounced and their origins are beginning to be understood (Jeanne, 1988; Jeanson and Weidenmüller, 2013). For instance, task specialization is seen in honeybees, and a colony even comprises both “employed” and “unemployed” foragers (Seeley, 1995). In bumblebees, there is some division of labour with smaller bees tending to the nest and larger bees devoting themselves more to foraging (Goulson et al., 2002). Foraging effort is anything but evenly shared (Free, 1955). Individual differences are not especially problematic in procedures where the number of unrewarded choices of stimuli is fixed. When bees are given unrestricted access to a flight cage, however, some bees will invariably make considerably more choices than others, and the issue arises as to their representativeness. A sample of undifferentiated ‘bee-choices’ (e.g. Lehrer et al., 1995) gives little guide as to whether the results might reflect the behaviour of only a few particularly active bees. Individual differences (e.g. Orbán and Plowright, 2013), colony differences (Plowright et al., 2011) and population differences (Skorupski et al., 2007; Ings et al., 2009) have been reported.

### Automation

Concerns over observer bias are common to many areas in the study of insect behaviour (Döring and Chittka, 2011), as are concerns over cost-effectiveness. The problems associated with human monitoring of flower-naïve bee behaviour, in real time, are compounded here because the occurrence of rare events over long observation periods almost inevitably leads to decreases in vigilance (Warm et al., 2009).

**Fig. 2:**
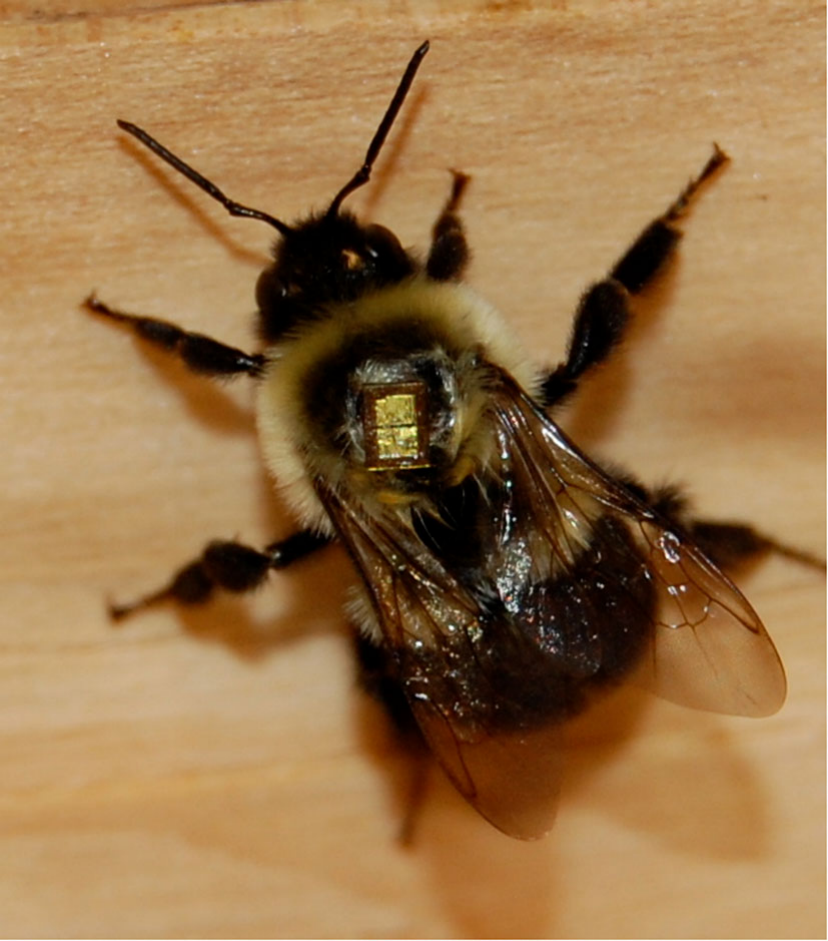
*B. impatiens* worker tagged with RFID chip. © L.L. Orbán

Two low-cost technological advances are now available: (1) while video recordings are commonplace (Leonard and Papaj, 2011), motion-sensitive camcorders (Lihoreau et al., 2012; Orbán and Plowright, 2013) have the added advantage of recording a specified length clip only when a specific pattern of movement is detected in the viewfinder. This feature is particularly well suited to this area of investigation because it filters out much of the time during which there is no activity around the stimuli. (2) Radio-frequency identification (RFID) is analogous to the bar-coding system that was pioneered by Buchmann for the identification of honeybees (the unpublished method is described by Reynolds and Riley (2002)). A metallic identification tag is glued to the thorax (see Fig. 2) and detected by a reader placed at strategic locations (Streit et al., 2003; Sumner et al., 2007; Ohashi et al., 2010; Stelzer and Chittka, 2010; Decourtye et al., 2011; Silcox et al., 2011; Nachev et al., 2012; Katzenberger et al., 2013). We have recently adapted the technology to detect flower-naïve bumblebees exploring unrewarding flowers (Orbán and Plowright, 2013). A video illustration of the procedure is shown by Orbán and Plowright (in press). One limitation of the method is the detection distance being restricted to a few millimetres (for other design considerations, see Carbunar et al., 2009). Electro-magnetic sensors to detect approach of flowers (Heuschen et al., 2005), used in conjunction with RFID, would be helpful in tracking behavioural sequences.

## Preferences of flower-naïve honeybees and bumblebees

**Fig. 3:**
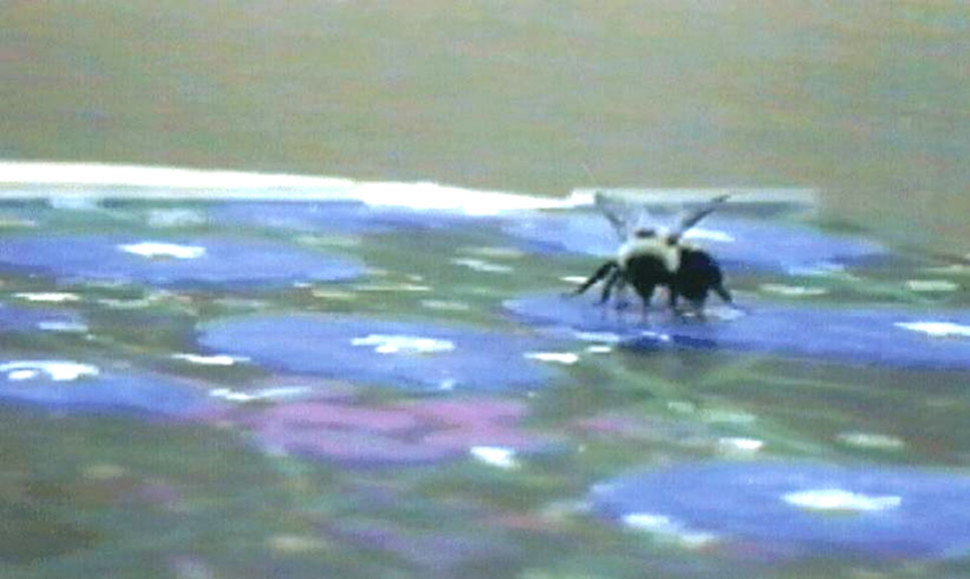
Flower-naïve bumblebee (*B. impatiens*) extending its proboscis towards a photograph of a flower. © V. Simonds. Photograph reproduced with permission

A casual observer who has ever seen a bee land on the floral picture on a seed packet or the floral print on an article of clothing may have had the distinct impression that the bee had been fooled: in the absence of discrimination training between flowers and pictures of flowers (Thompson and Plowright, 2014), the bees seem to have confused the two. A compelling demonstration is provided by Chittka and Walker (2006): bumblebees (*Bombus terrestris*) spontaneously land preferentially on Van Gogh’s *Sunflowers* rather than Caulfield’s *Pottery*. In our lab, we have seen a flower-naïve bumblebee probe a photograph of a flower (see Fig. 3). Here, we consider what might be the features of flowers that are particularly alluring.

### Colour

#### Colour perception

Unlearned colour preference is the most intensively studied floral visual property that includes the investigation of different frequencies of the electromagnetic spectrum, colour saturation, and contrast between patterns and backgrounds. Neurophysiological experiments show that bumblebees and honeybees have peak spectral sensitivities at approximately 350, 450 and 550 nm, which correspond to ultra-violet (UV), blue and green regions of the spectrum (Peitsch et al., 1992; Skorupski et al., 2007). There are no receptors with peak sensitivity near red, which likely accounts for poor learning of red (Chittka, 1997; Lunau et al., 2011), even in species (e.g. *Bombus dahlbomii* Guérin-Méneville, 1835) that are known to visit red flowers (Martínez-Harms et al., 2010). Colour vision functions only, however, at relatively short distances: up to 10 cm for a grating with a spatial period of 2 cm, or subtending an angle of 15° in honeybees, but only 2.7° in bumblebees (Lehrer et al., 1988; Land, 1997; Macuda et al., 2001; Chittka and Raine, 2006). Honeybees use green-contrast (i.e., grayscale vision) to an angle subtending up to 5° (2.3° for bumblebees) but beyond this point, the shapes of objects become indistinguishable (Dyer et al., 2008). The evolution of insect colour vision is reviewed by Briscoe and Chittka (2001).

**Fig. 4:**
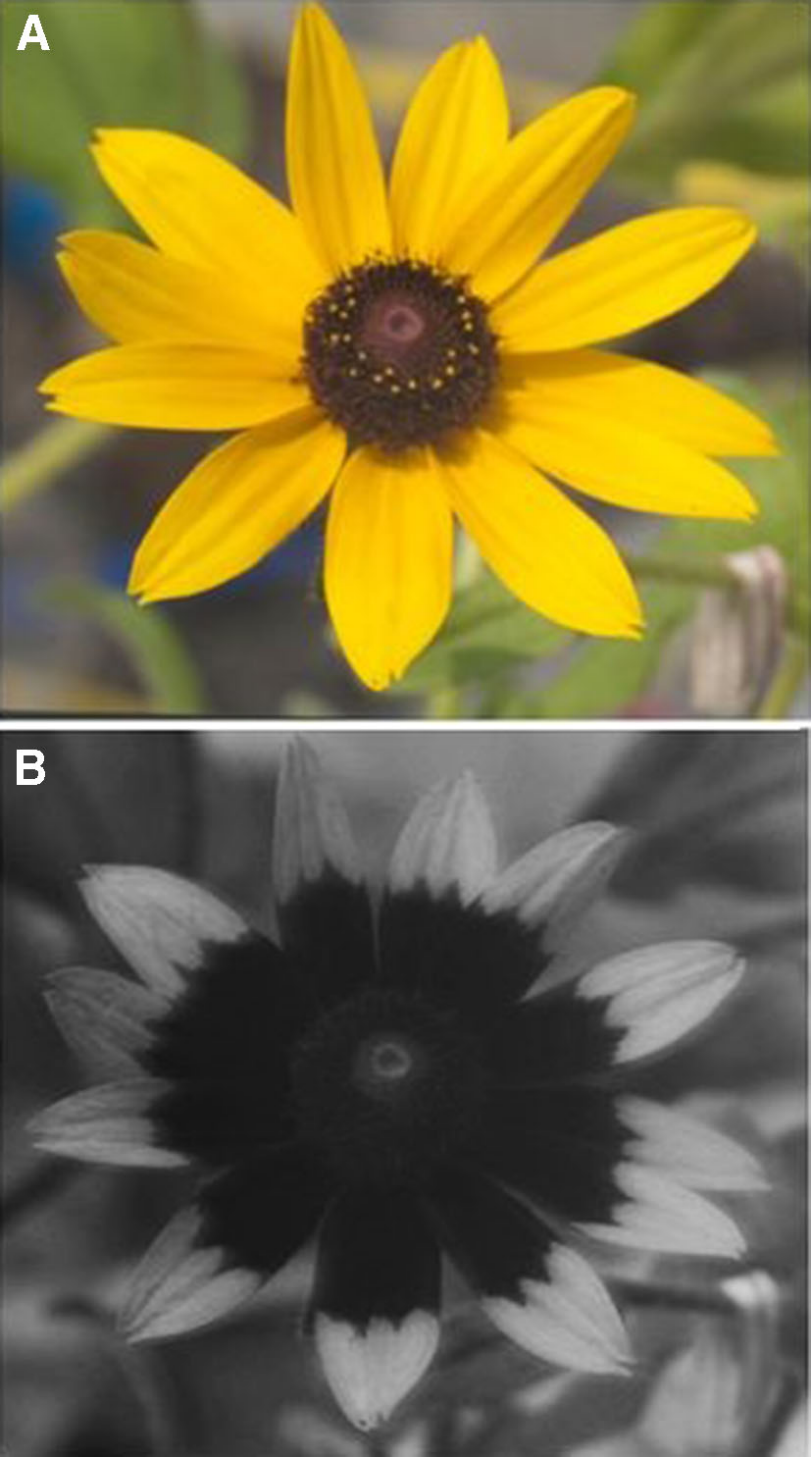
*Rudbeckia hirta* as seen with colour photography. **b** *R. hirta* as seen with ultraviolet (UV) photography. Photograph reproduced from an Open Access article (Horth et al., 2014) under a Creative Commons Attribution Licence

The fact that bees have sensitivity in the UV range of the spectrum is particularly important for plant–pollinator interactions. Ultraviolet absorbing “floral guides” that are invisible to humans are perceptible to pollinators. Not only do they serve to orient bees at close range towards the source of reward, but they also affect visitation rates (Horth et al., 2014). The appearance of flowers revealed by UV photography is illustrated in Fig. 4. In a manipulation of UV properties, Koski and Ashman (2014) have demonstrated that it is not the UV reflectance or absorbance alone but the patterns created on flowers that are attractive to pollinators.

The colour of nectar guides was studied on four spectral frequencies of 41 flower species: wavelengths of 360 nm (ultraviolet), 450 nm (blue), 520 nm (green) and 680 nm (red) (Penny, 1983). Flowers displayed nectar guides with better colour contrast on the insect visible spectrum (360 and 450 nm) when compared with the human visible spectrum (520 and 680 nm). The colour contrast effect was weaker when only UV was considered, suggesting that UV does not have a disproportionate contribution to preference: bees will choose yellow, and violet as well as UV. This behavioural finding about bee’s preference for several distinct colours is consistent with a study that showed non-UV flower colours are more common than UV flowers (Chittka et al., 1994), and another study that highlights the absence of pure UV flowers (Menzel and Shmida, 1993). Indeed, there is general agreement that the salience of floral UV patterns is comparable to the salience of other colours visible to bees (Kevan et al., 2001).

#### Floral colours

The colour preferences of both honeybees and bumblebees prior to any experience with flowers have been reviewed by Lunau and Maier (1995). While bumblebees and honeybees have similar colour vision (Peitsch et al., 1992), they differ in that honeybees have a highly developed communication system: experienced honeybees communicate the location of food sources to inexperienced bees (von Frisch, 1967). Nonetheless, honeybees (*A. mellifera*) with controlled prior experience (“neutral pre-training”) do have colour preferences (Giurfa et al., 1995) for wavelengths of 410 nm (“bee-uv-blue”) and 530 nm (“bee-green”): the same colours that are learned most easily (Menzel, 1967). Bumblebees have, at most, a primitive communication system (Dornhaus and Chittka, 1999, 2001) and individuals rely much more on their own efforts to find food.

The key dimension in triggering approach by untrained bumblebees and honeybees is not so much the dominant wavelength (giving rise to the perception of hue) of the corolla of a flower, but its spectral purity (giving rise to the perception of saturation), i.e. the degree to which there is one dominant vs. a mix of wavelengths (Lunau, 1990, 1992; Papiorek et al., 2013; Rohde et al., 2013). Corollas with high spectral purity are approached from afar (Lunau et al., 1996). Flowers incite inspection (*Bombus lucorum* (L., 1761) and *B. terrestris* (L., 1758)) with a gradient of spectral purity: low in the background, high at the corolla and highest at floral guides such as stamens. Spectral purity acts as a releaser for action patterns such as antennal reactions (Lunau, 1991) and other optical signals of stamens, such as size of the thecae and distance between them, elicit final landing (Lunau, 1991). Most flowers are not single-coloured, and indeed two-coloured flowers are preferred (Heuschen et al., 2005).

### Floral size

Under the principle that the evolution of floral signals is tied to pollinator perception, and that in nature floral size is possibly predictive of reward, Blarer et al. (2002) considered the possibility that there might be a preference for large flowers over smaller ones on the very first visit by bumblebees (*B. terrestris*). If such a preference were found, it would be important in terms of the evolution of floral displays: plants that honestly signalled their reward availability (see Armbruster et al., 2005) would be invasible by cheaters. Such a preference was not found, though with experience, bees were capable of associating floral size with reward (Blarer et al., 2002). In a more recent study, we used artificial flowers that consisted of two blue perpendicular acrylic sheets perched on top of a container that trapped bees that entered. The design of these flowers was based on traps used in the field to census insect populations (Stephen and Rao, 2005). We manipulated the size of the flowers, but no effect on the choices of flower-naïve bumblebees (*B. impatiens*) was detected (Hudon and Plowright, 2011). The usual cautions in interpreting failures to reject the null hypothesis apply.

### Patterning

#### Floral guides

The suggestion that floral markings function to guide pollinators towards the nectary likely originated with Sprengel (1793). When patterns are presented at the ends of corridors in a maze, selective approach of radial patterns (i.e. ‘sunburst’ patterns: alternating black and white pie shaped segments, all pointing to the centre) over concentric patterns (i.e. ‘bull’s eye’ patterns: alternating black and white circles within each other) has been well documented for honeybees (Lehrer et al., 1995) and bumblebees (Simonds and Plowright, 2004; Plowright et al., 2006; Séguin and Plowright, 2008). Selective landing on radial patterns by free flying bumblebees has also been reported (Orbán and Plowright, 2013). Whether flowers have petals or not seems comparatively unimportant: it is the presence of radial lines on artificial flowers that causes bumblebees not only to make their first landing but also to locate food more quickly after landing (Leonard and Papaj, 2011). These lines are beneficial for the plants: they discourage nectar robbing (Leonard et al., 2013). Experimental removal of “floral signposts” has a detrimental effect on plant fitness (Hansen et al., 2012; Whitney et al., 2013).

While the evidence above shows that “X marks the spot” (Leonard and Papaj, 2011), other shapes also seem to be used as floral guides. Flowers with a dot or pair of dots at the centre of flower (Fig. 1) are more likely to be approached and antennated by flower-naïve bumblebees (Heuschen et al., 2005), with bigger dots eliciting stronger responses by both honeybees and bumblebees (Lunau et al., 2009).

#### Pattern location

The case for special markings functioning as guides to the nectary or to the anthers is bolstered by a recent experiment where the presence of the food source was dissociated from the place indicated by the guide. When the position of the nectary conflicted with the ‘directions’ given by an off-centre guide, unsuccessful novice bumblebee foragers spent significantly more time searching for nectar than when the nectar guides surrounded the nectary (Goodale et al., 2014). Using a similar experimental strategy of dissociating two variables, pattern type (radial vs. concentric) and location of the pattern elements (central vs. peripheral), we showed that both concentric elements and radial elements caused bumblebees to enter an artificial flower, as long as the elements were centrally located: both ‘X’ and ‘O’ marked the spot. Concentric elements at the periphery of the flower put bumblebees that had landed on the flower on a circular path that steered them clear of the centre. Landing was more likely on artificial flowers displaying radial elements, regardless of whether they were positioned centrally or peripherally (Orbán and Plowright, 2013).

#### Spatial frequency

In nature, some plants make themselves detectable not by their particularly large floral structures, but by their inflorescences consisting of clusters of small flowers (Lehrer et al., 1995): spatial frequency (i.e. the “busyness”) of the visual input may be a key variable. Honeybees show a gradient of strong to weak preference for clusters consisting of four, three, two or one radiating patterns. In comparisons of various spatial frequencies for each of several patterns (horizontal gratings, vertical gratings, radial patterns and concentric circles), however, a consistent preference for comparatively low frequencies was obtained, even though the most disrupted patterns were resolvable—i.e. the lines were not perceived as blurred together (Lehrer et al., 1995). Preferences for relatively high spatial frequency patterns have also been reported (Dafni et al., 1997; Plowright et al., 2011). Several possible explanations might account for the discrepancies across studies: (1) the absolute values for spatial frequencies likely differ across studies, with “high” and “low” being relative terms. (2) It is not so much the spatial frequency per se that is important, as it is the associated contrast with the background, as suggested by Lehrer et al. (1995)—indeed, even colour preferences of bumblebees are affected by background complexity (Forrest and Thomson, 2009). (3) As suggested below, the effect of spatial frequency may depend on another variable: symmetry.

#### Symmetry

An important consideration with regard to what draws bees to flowers for the first time is how easy it is to encode and remember a floral pattern should it turn out to be rewarding. In other words, perceptibility, learnability and memorability of the pattern may turn out to be important aspects of what makes flowers attractive to bees. Indeed, Nachev (2014) has recently made the case for “cognition mediated evolution”. This consideration puts the study of floral preferences squarely in the domain of psychology. Cognition, perception, neuroscience and computational modelling are here at centre stage.

The evolution of floral symmetry has been reviewed by Neal et al. (1998). Preferences for floral symmetry have been documented in the field. Naturally occurring symmetric flowers of fireweed (*Epilobium angustifolium*) were preferentially visited by *B. terrestris*, and this was also true of experimentally manipulated flowers that affected symmetry (Møller, 1995; see also Møller and Sorci, 1998). In the lab, honeybees perceive symmetry, as evidenced by their ability to learn discriminations between symmetric and asymmetric patterns and to generalize this learning to novel patterns (Giurfa et al., 1996). The evidence on a preference for symmetry by flower-naïve bees, however, is mixed. No such preference was reported by West and Laverty (1998), though bumblebees could learn that symmetric flowers were rewarding just as easily as they could learn that asymmetric flowers were rewarding. An “innate” preference for symmetry about the vertical axis (i.e. bilateral symmetry) on vertically presented flowers (so the line of approach was perpendicular to the plane on which the pattern was presented) by bumblebees was reported by Rodríguez et al. (2004). It seems, however, to have been the product of pre-training on rewarding discs (Plowright et al., 2011): truly flower-naïve bumblebees showed no preference for bilateral symmetry in vertically presented flowers. More recently, however, a preference for symmetry was found by increasing the strength of the manipulation: patterns for which there were four axes of symmetry, and not just one, were indeed chosen over asymmetric patterns (Orbán, 2014).

Symmetry in flowers may well be an index of floral reward (Møller and Eriksson, 1995). Symmetry also affords considerable savings in terms of information processing since part of the pattern (half or even more, depending on the number of axes of symmetry) can be discarded without losing any information to be remembered. The cost of information processing may translate into metabolic costs (Laughlin et al., 1998) and bees may act to minimize these costs as they search for flowers. A key point is that symmetry simplifies the processing of a complex pattern, and low spatial frequency simplifies the processing of an asymmetric pattern: the effect of one variable should depend on another. This notion of computational savings was captured in a mathematical model of pattern reconstruction (ICA: Independent Component Analysis; Orbán and Chartier, 2013). The essence of ICA is that the visual system completes a process akin to a dimensionality reduction process whereby the raw visual input is reduced to a small set of descriptive features. The model made novel predictions that were borne out empirically. For instance, a preference for low spatial frequency patterns over high-frequency patterns was found, but this preference was only detected when the patterns were ‘cumbersome’ by virtue of being asymmetric (Orbán, 2014).

### Social cues

Up to now we have been considering aspects of flowers that elicit initial choice. Recent research has addressed the question of whether the presence of foragers on flowers functions the same way as floral properties: perhaps an individual on a flower attracts other bees towards it. From a mechanistic point of view, local enhancement or stimulus enhancement, whereby one individual attracts another to a particular location or stimulus, is commonplace in animal behaviour (Shettleworth, 2010). From a functional point of view, however, such a possibility is only one of other plausible scenarios. Perhaps in nature floral characteristics are such strong predictors of reward that additional social cues carry little additional informational value. Another possibility is that while the presence of a forager signals that a flower has indeed been discovered, it also signals that the flower is empty or on its way to being depleted. In other words, other foragers may act as informers or as competitors (Baude et al., 2011). In view of these considerations, it might be expected that the predictive value of the presence of other foragers on flowers might depend on local environmental conditions and might only be learned from experience. We turn now to the evidence on this point.

Recent reports have shown that a preference for “occupied” flowers is not only modified by rewarded experience (Leadbeater and Chittka, 2009; Avarguès-Weber and Chittka, 2014), but is also apparent as soon as bees first begin to search for food. Inexperienced bumblebees given a choice between two rewarding artificial flowers, one of which was occupied by a dead pinned bee and the other not, first landed on the occupied flower more frequently than chance (Kawaguchi et al., 2006). Similarly, inexperienced bumblebees given a choice amongst 12 unrewarding artificial flowers, four of which were occupied by a dead pinned bee and eight of which were not, first landed on an occupied flower more frequently than chance (Leadbeater and Chittka, 2009). A preference for occupied stimuli was also found by Plowright et al. (2013), but only under a restricted set of conditions: when the occupied flowers were comparatively rare, and in addition, the ratio of the size of the occupier relative to the size of the flower was comparatively large. Otherwise, choice proportions did not differ from chance.

Little is known about how the presence of other foragers is perceived by bees making floral choices. They may be possibly perceived as being parts of the flowers such as nectar guides (Baude et al., 2008), or they may be perceived as other inanimate objects such as a coin or a plastic disc (Dawson and Chittka, 2012). There is evidence that flowers have adapted their visual appearance to exploit the salience provided by the presence of other foragers. For example, a South-African daisy species (*Gorteria diffusa*) displays insect-mimicking petal spots (Thomas et al., 2009; Whitney et al., 2011).

## Conclusion

Bees discover all kinds of flowers that have few similarities. Lilacs (*Syringa vulgaris*), comfrey (*Symphytum officinale*), monkshood (*Aconitum* spp.), thistles (*Cirsium* spp.), blueberry and cranberry flowers (*Vaccinium* spp.), sunflowers (*Helianthus annus*) and tomato flowers (*Solanum lycopersicum*) do not share all the same colour, contrast, symmetry, spatial frequency or size properties. Moreover, these flowers do share at least some features with other objects that are not flowers (e.g., leaves are usually symmetric; some insects reflect UV). This review has shown that several floral properties are attractive to honeybees and bumblebees with no previous foraging experience, but there seems to be no single set of essential features that define the category of “food source” or even “possibly a food source”.

There is no shortage of problems and unanswered questions to address, of which we enumerate a few here for consideration in future research:

1. The question of the nature of experience, i.e. how the bee sees the world, remains open. Cautions against anthropomorphism abound, but they bear repeating. Not only do flowers that look the same to humans look different to bees by virtue of their UV patterns, but the reverse is also true: Dyer et al. (2007) have shown that two variants of snapdragon (*Antirrhinum majus*) look very different to humans but are treated as the same by bumblebees.
2. The question of how to bridge the gap between the lab studies delineated here and behaviour in the considerably larger scale environment in the field is also as worthy of investigation for bees as it is for other animals (Shettleworth, 1989).
3. There are about 250 *Bombus* species worldwide (Williams and Osborne, 2009) and yet the research reviewed above has focused on a handful of easily available species such as *B. impatiens* and *B. terrestris*. There are fewer than ten *Apis* species, but one of them, *Apis mellifera*, has been over-represented in the research. Flower visitors specialize on certain plant traits (Junker et al., 2013) and generalizations based on a few species are almost certainly limited.
4. Our list of important visual cues, used in isolation or in conjunction with other cues, will likely expand with future research. Just recently, the use of polarization patterns by *B. terrestris* has been demonstrated in learned discriminations between artificial flowers (Foster et al., 2014). In the past, polarization had only been known to be important in navigation (Rossel, 1993).
5. The testing for the effect of variables one by one for their value as releasers is inefficient. A more contemporary approach would be to determine how bees classify multi-dimensional signals (Shettleworth, 2010). For instance, floral “salience”, which is a function of the intensity of several floral stimuli, turns out to be a parsimonious explanatory variable (Katzenberger et al., 2013).
6. Our purpose here was most certainly not to dissect behaviour into categories. It was to put the focus on the precursors of behaviour learned from experience with flowers: the scaffolding on which learning is built. These behaviours are likely to be important from a conservation point of view: though possible disruptions in how bees first find food may turn out to be inconsequential, it seems an unlikely scenario. Ultimately, however, the goal is to understand the development of functional behaviour. Future research should be aimed at linking what we know about the behaviours of comparatively inexperienced workers with what we know about experienced workers. Several recent studies have investigated the fate of the preferences that guide bees to their first floral contact: How easily are they forgotten (Milet-Pinheiro et al., 2012)? Are they distracting (Morawetz et al., 2013)? Can they be associated with consequences such as rewards or punishers (Pohl et al., 2008)? Given the current research effort aimed at protecting pollinators in general, at protecting bees in particular, and especially at understanding ‘the plight of the bumblebee’, none of these questions are idle.
